# Metagenomic next-generation sequencing aids the diagnosis of viral infections in febrile returning travellers

**DOI:** 10.1016/j.jinf.2019.08.003

**Published:** 2019-10

**Authors:** Hanna Jerome, Callum Taylor, Vattipally B. Sreenu, Tanya Klymenko, Ana Da Silva Filipe, Celia Jackson, Chris Davis, Shirin Ashraf, Eleri Wilson-Davies, Natasha Jesudason, Karen Devine, Lisbeth Harder, Celia Aitken, Rory Gunson, Emma C. Thomson

**Affiliations:** a MRC-University of Glasgow Centre for Virus Research, Sir Michael Stoker Building, 464 Bearsden Road, Glasgow G61 1QH, UK; b Department of Infectious Diseases, Queen Elizabeth University Hospital, 1345 Govan Rd, Govan, Glasgow G51 4TF, UK; c West of Scotland Specialist Virology Centre, Level 5, New Lister Building, Glasgow Royal Infirmary, 10-16 Alexandra Parade, Glasgow G31 2ER, UK; d Queen Elizabeth University Hospital, 1345 Govan Rd, Govan, Glasgow G51 4TF, UK

**Keywords:** Returning travellers, NGS, Diagnosis

## Abstract

**Objectives:**

Travel-associated infections are challenging to diagnose because of the broad spectrum of potential aetiologies. As a proof-of-principle study, we used MNGS to identify viral pathogens in clinical samples from returning travellers in a single center to explore its suitability as a diagnostic tool.

**Methods:**

Plasma samples from 40 returning travellers presenting with a fever of ≥38°C were sequenced using MNGS on the Illumina MiSeq platform and compared with standard-of-care diagnostic assays.

**Results:**

In total, 11/40 patients were diagnosed with a viral infection. Standard of care diagnostics revealed 5 viral infections using plasma samples; dengue virus 1 (*n* = 2), hepatitis E (*n* = 1), Ebola virus (*n* = 1) and hepatitis A (*n* = 1), all of which were detected by MNGS. Three additional patients with Chikungunya virus (*n* = 2) and mumps virus were diagnosed by MNGS only. Respiratory infections detected by nasal/throat swabs only were not detected by MNGS of plasma. One patient had infection with malaria and mumps virus during the same admission.

**Conclusions:**

MNGS analysis of plasma samples improves the sensitivity of diagnosis of viral infections and has potential as an all-in-one diagnostic test. It can be used to identify infections that have not been considered by the treating physician, co-infections and new or emerging pathogens.

**Summary:**

Next generation sequencing (NGS) has potential as an all-in-one diagnostic test. In this study we used NGS to diagnose returning travellers with acute febrile illness in the UK, highlighting cases where the diagnosis was missed using standard methods.

## Highlights

• Next generation sequencing (NGS) has potential as an all-in-one diagnostic test for infection.
• Viral, bacterial and parasite genomes can be detected rapidly.
• NGS is becoming increasingly affordable but requires validation in clinical laboratories.

## Introduction

The ease of international travel has increased the potential for transmission of a wide range of viruses, including those of epidemic potential. Identifying these pathogens is crucial for treatment and for the prevention of further transmission. Traditional diagnostic tests require *a priori* knowledge of potential pathogenic agents and it is increasingly challenging for infectious disease physicians to select relevant diagnostic tests for pathogens imported from a wide number of locations across the globe.^1^ Such tests are often batched and sent to a reference laboratory, often resulting in delayed diagnosis. Even specialized tests may fail to identify a rapidly evolving pathogen, due to the presence of variations at PCR primer binding sites. Furthermore, new and emerging pathogens cannot be identified using standard assays. In contrast, new or emerging infections and those not considered by a treating physician may readily be identified with MNGS.^2^, ^3^, ^4^, ^5^, ^6^ Whole viral genome sequences generated using MinION or Illumina-based technology additionally be used for resistance screening and fine-scale phyloepidemiological analyses to inform public health control strategies.^7^, ^8^ In this study, we aimed to evaluate the potential for MNGs as an all-in-one diagnostic tool in febrile patients with a recent history of overseas travel.

The value of MNGS as a tool to identify pathogens not identified by traditional methods has been described in case reports of patients infected with a number of pathogens. One notable example involved the use of MNGS to diagnose a 14-year old boy with neuroleptospirosis, resulting in a change of management to incorporate appropriate antibacterial therapy.^9^ It has also been used in outbreaks of viral haemorrhagic fever (VHF) where standard diagnostic assays did not reveal a diagnosis.^5^, ^10^ A novel strain of Ebola virus, Bundibugyo virus, not readily identified using standard assays was identified following metagenomic 454 pyrosequencing of patient samples in 2008 during an outbreak of VHF in Western Uganda.^5^ 454 pyrosequencing and subsequently Illumina sequencing were used in another outbreak of viral haemorrhagic fever in the Democratic Republic of the Congo and revealed a novel rhabdovirus – Bas Congo virus – as the aetiological agent.^10^ Causes of VHF had previously included members of the Filovirus and Arenavirus families but not the Rhabdovirus family and therefore pathogen-specific diagnostic assays would have been unhelpful. Without the use of MNGS, it is unlikely that this pathogen would have been identified.

A number of NGS platforms have been used to diagnose viral pathogens, based on different technologies.^11^ These are divided into short and long-read methods. At present, short read methods are higher-throughput and cheaper than long-read methods. Short read methods divide into sequencing by ligation and sequencing by synthesis. 454 pyrosequencing (in which the release of a pyrophosphate is incorporated in a sequencing by synthesis reaction that results in the release of light) was the first available short read NGS platform but is limited by a high error rate (largely due to polynucleotide repeats). Illumina sequencing is also a short-read sequencing by synthesis method and is based on the high-throughput detection of fluorescent markers as they are incorporated into an expanding chain. It is cheaper and has a higher level of accuracy allowing accurate sequencing of genomes from new pathogens and for fine-scale phyloepidemiology. It is limited by sequence read lengths of around 500 base pairs, requiring advanced bioinformatic software to reconstruct viral genomes. Longer sequence reads may be acquired on other platforms such as the PacBio, the Oxford nanopore and the Ion Torrent platforms that are based on semiconductor technology (due to a change in ionic charge when a base is added to the expanding chain). These technologies are limited however by a decreased accuracy when compared with Illumina-based methods. A particularly exciting advance has been the development of the MinIon platform which is portable, the size of a mobile telephone and can be used in the field without an external internet or power source.^12^ The MinIon platform has been used recently to track the Zika virus outbreak in South America and Ebola virus disease in the Democratic Republic of Congo.^12^, ^13^ It is limited in accuracy however and in the need for high concentrations of input DNA necessitating prior amplification of genomic material by PCR but has potential for further development as a diagnostic tool if high error rates can be addressed.

## Methods

### Patient consent and sample collection

The retrospective use of samples from patients admitted with a febrile illness following overseas travel between 2013 and 2016 was granted by the NHSGGC Bio-repository and Pathology Tissue Resource committee (16/WS/0207NHS). Samples were anonymized and patients who had not had an HIV test during admission were excluded (to avoid the possibility of diagnosing a new HIV infection using MNGS). Inclusion criteria were a fever greater than 38°C, age ≥18 years, a preceding negative HIV test and overseas travel within 12 weeks of presentation to hospital. 44 samples from 40 patients were available for MNGS analysis. Diagnoses were made by two blinded investigators (an infectious diseases consultant physician and a senior laboratory scientist with expertise in NGS data analysis) prior to comparison with clinical data.

### Extraction of DNA and RNA

RNA and DNA were extracted from 200 µl of serum or plasma samples using the automated NucliSens EasyMAG platform at the West of Scotland Specialist Virology Centre, Glasgow Royal Infirmary. Nucleic acid was eluted in 110 µl nuclease-free water and stored at −80°C until further processing.

### Double stranded cDNA synthesis

RNA was reverse transcribed into cDNA using Superscript III reverse transcriptase (Invitrogen) according to manufacturer's recommendations. The total reaction volume from single-stranded cDNA synthesis (20 µl) was used to create double stranded cDNA with a New England Biolabs Second Strand cDNA synthesis kit according to manufacturer's recommendations in a final volume of 80 µl.

### Library preparation and MiSeq sequencing

For library preparation either the Kapa Library Preparation Kit (KK8232 Kapa Biosystems) or the Nextera XT Kit (Illumina FC-131-1024) was used in combination with the NEBNext® Multiplex Oligos for Illumina® (Index Primers Set 1 and 2, E7335 and E7500) or the Nextera® XT Index Kit (FC-131-2001). To estimate the molarity of DNA libraries after indexing PCR, DNA concentration was measured using the High Sensitivity double-stranded DNA Qubit Assay (Invitrogen), and the quality of the DNA was assessed using automated gel electrophoresis using a 2200 TapeStation Instrument (Agilent Technologies). For DNA purification between the library preparation steps, Agencourt® AMPure® XP magnetic beads (Beckman Coulter) and Solid Phase Reversible Immobilization solution (SPRI, 20% PEG, 2.5 M NaCl) were used, respectively. Up to 20 DNA libraries were pooled together for 150 or 250 paired end sequencing using v2 or v3 cartridges (Illumina) on the MiSeq or NextSeq platform according to manufacturer's recommendations.

### Bioinformatic analysis

Sequences of low quality and complexity and Illumina adaptor sequences were removed from raw fastq files using Trim_galore (Babraham Bioinformatics) and Prinseq.^14^ Raw reads were used to carry out a blastx search using diamond (version 0.8.20) software. Subsequently, *de novo* assembly and mapping approaches were applied using SPAdes-3.8.0,^15^ dipSPAdes^16^ and Tanoti (version 1.3, https://github.com/vbsreenu/Tanoti). Contigs were identified using BLASTn and BLASTx (blastall version 2.2.26) against ncbi nr and nt databases and diamond databases.^17^ The output was visualized using Krona plots and BLAST tables.^18^ Additionally, a rapid hash-based exploration tool was created and evaluated (Anwesh). This software applies BLAT to screen MNGS datasets with customized curated databases of nucleotide sequences. Two databases were created for this purpose, derived from NCBI (ftp://ftp.ncbi.nlm.nih.gov/genomes/Viruses/) and the ‘Approved List of biological agents’ published by the UK Health and Safety Executive (HSE). When a match to a sequence was detected during screening with Anwesh or by *de novo* assembly, this was used for mapping using Tanoti. As MNGS is subject to low-level cross-contamination, we used a threshold of a minimum of coverage of 10% of the viral genome for diagnosis and repeated testing from a run where a sample was positive with a higher number of mapped reads (Supplementary Fig. 1). This threshold was chosen as it approximates the size of PCR product used in routine diagnostic assays.

#### Confirmatory testing

Confirmatory tests were carried out by PCR or serology in the West of Scotland Specialist Virology Centre (WoSSVC), Glasgow Royal Infirmary or sent to the Rare and Imported Pathogens Laboratory (RIPL, Public Health England Porton Down).

## Results

**Fig. 1:**
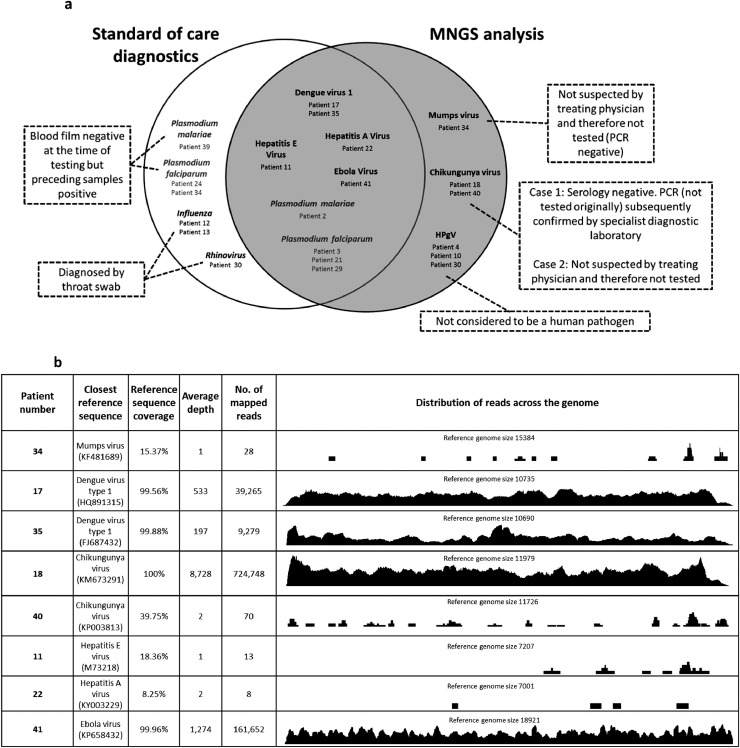
Viral and parasitic diagnoses using standard of care and MNGS testing. (a) Viral and parasitic diagnoses from plasma samples derived from returning travellers using standard of care diagnostics (white circle) and blinded retrospective MNGS analysis (gray circle). (b) Genome coverage, depth, number and distribution of mapped sequence reads.

Plasma samples from 40 febrile returning travellers and 1 afebrile control patient were analyzed using MNGS (Fig. 1). Relevant travel histories and clinical and standard of care laboratory diagnosis are listed in Supplementary Table 1. MNGS analysis resulted in the detection of 8 viral genomes: Dengue virus 1 (DENV1; *n* = 2; P17 and P35), Chikungunya virus (CHIKV; *n* = 2; P18 and P40), hepatitis E virus (HEV; *n* = 1; P11), hepatitis A virus (HAV; *n* = 1; P22), mumps virus (*n* = 1; P34) and Ebola virus (EBOV; *n* = 1; P41) (Fig. 1(a)). A range of depth and coverage of viral read results were identified (likely reflecting a variety of viral loads) (Fig. 1(b)). Three patients who were infected with respiratory viruses based on viral throat swab testing – P12 (influenza A virus), P13 (influenza B virus), and P30 (rhinovirus) could not be fully evaluated by MNGS because respiratory samples were not available for analysis. Additionally, human pegivirus (HPgV) was detected in 3 patients (patients P4, P10 and P30). This is not currently considered to be a human pathogen. These were all genotype 2 and one strain was highly divergent, nearly meeting the criteria for a new genotype based on current ICTV recommendations (Supplementary Fig. 2). While the focus of this project was the detection of viral infections, we also detected *Plasmodium falciparum* and *Plasmodium malariae* genomic material in patients P2, P3, P21 and P29. Three patients (P24, P34 and P39) were found to have malaria on blood film sampling but not NGS (in each case the date of viral sampling for NGS occurred subsequent to blood film positive results). One of these patients (P34) had been diagnosed previously with *P. falciparum* malaria on blood film and had sampling for other pathogens because of ongoing fever and lymphadenopathy despite therapy – fragments of mumps virus genome were detected in this sample. Subsequent PCR testing by the diagnostic laboratory was negative.

**Fig. 2:**
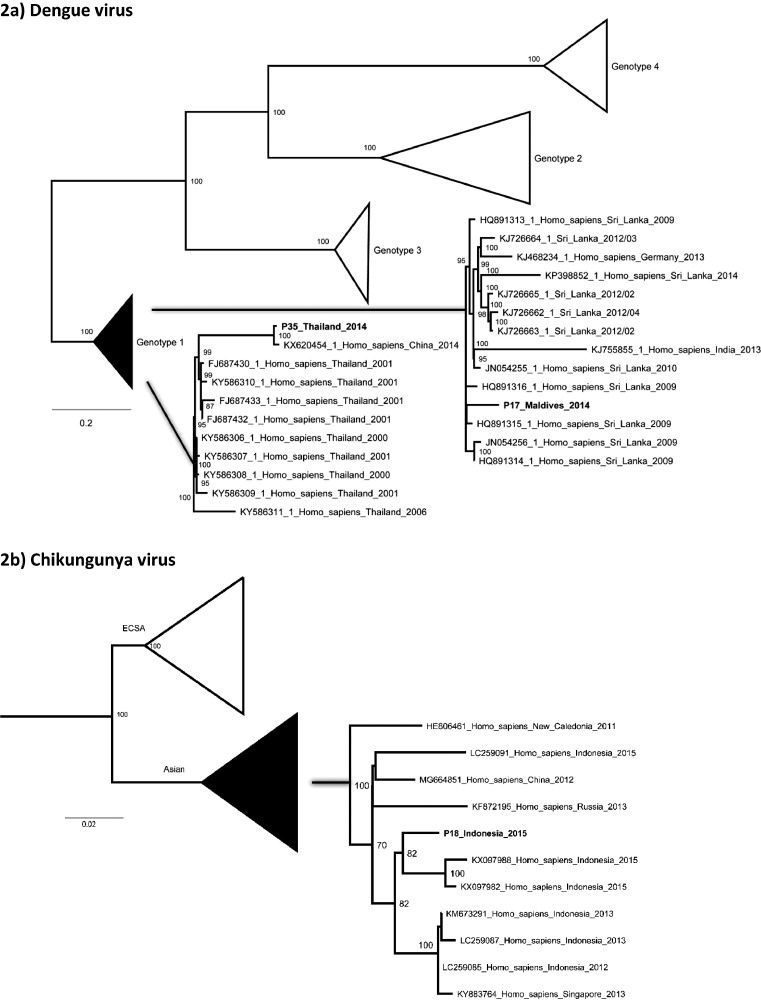
Phylogenetic analysis of whole viral open reading frames detected using NGS. Maximum likelihood phylogenetic analysis of patient samples and all available full genome reference sequences (NCBI) was carried out using RA×ML and 1000 bootstrap replicates.

Maximum likelihood phylogenetic analysis of DENV and CHIKV full genomes (Fig. 2) revealed that the CHIKV viral genome from P18 clustered within the Asian strain of CHIKV and was most closely associated with other CHIKV strains from Indonesia in keeping with the travel history of the patient. Phylogenetic analysis of the DENV strains revealed that P35, a returning traveller from Thailand had a strain clustering with genotype 1 strain from China and Thailand while P17, a returning traveller from the Maldives had a strain most closely related to strains from Sri Lanka (this is the first published DENV whole genome sequence from the Maldives). Whole genomes detected in the study have been submitted to GenBank (accession numbers to follow).

## Discussion

MNGS has the potential to improve the diagnostic yield of viral, bacterial and parasitic infectious diseases.^2^^,^^19^, ^20^, ^21^, ^22^, ^23^, ^24^, ^25^, ^26^, ^27^, ^28^ Importantly, it does not require the requesting physician to consider all diagnostic possibilities when ordering the test; in our small proof-of-concept study, 3 additional viral diagnoses were made using MNGS (Chikungunya virus in two cases and mumps virus in one). Furthermore, MNGS has the potential to detect multiple pathogens in a single sample. In our study, one patient (34) who had been admitted with *Plasmodium falciparum* infection but was noted to have prominent cervical lymphadenopathy and ongoing fever despite treatment was found to have fragments of mumps virus following MNGS. This was in keeping with ongoing symptoms of lymphadenopathy and fever despite negative blood films (*P. falciparum* was not detected in this case, but at the time of sampling, the blood film was noted to be negative, having been positive at an earlier time point). Full genome data generated by MNGS has other potential applications – for example, identification of virulence genes or resistance mutations including as minority variants and the identification of clusters of infection using phylogenetic approaches. Infections of consequence can also be identified and relevant treatment and public health precautions carried out in real time to aid the treatment of an affected patient.^29^ In this study, one returning traveller presented with a relapse of Ebola virus disease associated with meningoencephalitis, 10 months after her original presentation. In this case, the full open reading frame of the virus was sequenced in real time both in plasma and cerebrospinal fluid in order to facilitate experimental treatment selection and was found to be identical in both sites on relapse. This was the first evidence of EBOV causing disease in the central nervous system and the only available sequence obtained from cerebrospinal fluid – a site not previously considered to harbor the virus.

There are a number of limitations to this study. Firstly, our method is designed to detect RNA viruses (the majority of viruses associated with travel are RNA viruses) and employs a DNAse step to reduce sequencing human genomic material. It is likely to detect DNA viruses with an RNA stage in the life cycle but with reduced sensitivity. To increase the detection threshold for DNA viruses, a different approach such as target enrichment (allowing separation of viral from host DNA) or removal of methylated DNA should be considered. Secondly, we did not have the opportunity to test respiratory, CSF and urine samples in the majority of cases; however others have identified novel viral pathogens such as astroviruses using other body fluids and tissues.^20^, ^25^, ^30^ Thirdly, we have not formally measured sensitivity of MNGS for the detection of each pathogen identified in this study. We have previously evaluated the threshold for detection of the hepatitis C virus and MNGS can reliably allow reconstruction of a full HCV genome above a threshold of 10,000 IU/ml using MNGS and 1000 IU/ml using target enrichment approaches.^7^ Finally, we did not design this study to include analysis of bacterial genomes. Further validation of this technique and the evaluation of detection sensitivity will be required as the method is rolled out.

There are still barriers to overcome before MNGS is implemented as a routine diagnostic assay. Firstly, MNGS is highly subject to cross-contamination and therefore stringent controls must be in place to manage the pipeline (separation of preparation rooms at each step of the sequencing pathway and strict bioinformatic thresholds). Secondly cost considerations must be made. At the time of writing, the cost of MNGS is approximately $100 USD/sample – this is not much higher than the cost of individual tests requested in febrile patients from centralized specialist centers but will require consideration of batching and may only be feasible in major centers with a high throughput of patients. Formal cost-effectiveness and validation studies are required.

At the current time, the Illumina platform has the highest accuracy, but this may not be essential when using the tool simply to make a diagnosis. The portable MinIon platform has the potential as a bedside test but requires further development of library preparation platforms that are also portable and a reduction in the high amount of starting material required at present.^31^, ^32^ Inaccuracy in sequence reads may not be of major importance if diagnosis is the main goal, particularly with viruses where genomes are extremely diverse, differing from another by more than 30%.

There is potential for MNGS to provide a single test to diagnose pathogens that may be viral, parasitic or bacterial on the basis of specific syndromic manifestations, for example, returning travellers with fever. However, wet lab techniques and bioinformatic methods will need to be adapted to issues associated with different pathogens. Diagnosis of bacterial infections using MNGS may be best carried out following culture or 16S PCR. The potential for contamination from bacteria on the skin surface must be considered but is a lesser problem when identifying viral or parasitic pathogens. Malaria is an essential consideration in returning travellers but may require adaptations of sample processing – for example the use of whole blood would be likely to increase the sensitivity of detection of blood stage parasites but would carry the associated need to carry out more sequencing due to dilution of samples with host DNA.^33^, ^34^ It is even possible that a single sample could yield information on host mutations rendering the patient more or less susceptible to certain agents (for example CCR5 deficiency is likely to provide protection against the HIV virus but susceptibility to tick-borne encephalitis virus, West Nile virus and influenza).^35^, ^36^ However, the development of such a test will require specialists from multiple specialities to co-ordinate and work together to develop appropriate approaches to diagnosis. It is an aim well-worth pursuing.

## Declaration of competing interest

None.

## Appendix Supplementary materials

Image, application 1

## References

[1] Dong J., Olano J.P., McBride J.W., Walker D.H. (2008). Emerging pathogens: challenges and successes of molecular diagnostics. J Mol Diagn.

[2] Wilson M.R., Sample H.A., Zorn K.C., Arevalo S., Yu G., Neuhaus J. (2019). Clinical metagenomic sequencing for diagnosis of meningitis and encephalitis. N Engl J Med.

[3] Briese T., Paweska J.T., McMullan L.K., Hutchison S.K., Street C., Palacios G. (2009). Genetic detection and characterization of Lujo virus, a new hemorrhagic fever-associated arenavirus from southern Africa. PLoS Pathog.

[4] Hoffmann B., Tappe D., Hoper D., Herden C., Boldt A., Mawrin C. (2015). A variegated squirrel bornavirus associated with fatal human encephalitis. N Engl J Med.

[5] Towner J.S., Sealy T.K., Khristova M.L., Albarino C.G., Conlan S., Reeder S.A. (2008). Newly discovered Ebola virus associated with hemorrhagic fever outbreak in Uganda. PLoS Pathog.

[6] Chiu C.Y., Miller S.A. (2019). Clinical metagenomics. Nat Rev Genet.

[7] Thomson E., Ip C.L., Badhan A., Christiansen M.T., Adamson W., Ansari M.A. (2016). Comparison of next-generation sequencing technologies for comprehensive assessment of full-length hepatitis C viral genomes. J Clin Microbiol.

[8] Quick J., Loman N.J., Duraffour S., Simpson J.T., Severi E, Cowley L. (2016). Real-time, portable genome sequencing for Ebola surveillance. Nature.

[9] Wilson M.R., Naccache S.N., Samayoa E., Biagtan M., Bashir H., Yu G. (2014). Actionable diagnosis of neuroleptospirosis by next-generation sequencing. N Engl J Med.

[10] Grard G., Fair J.N., Lee D., Slikas E., Steffen I., Muyembe J.J. (2012). A novel rhabdovirus associated with acute hemorrhagic fever in central Africa. PLoS Pathog.

[11] Goodwin S., McPherson J.D., McCombie W.R. (2016). Coming of age: ten years of next-generation sequencing technologies. Nat Rev Genet.

[12] Quick J., Grubaugh N.D., Pullan S.T., Claro I.M., Smith A.D., Gangavarapu K. (2017). Multiplex PCR method for MinION and Illumina sequencing of Zika and other virus genomes directly from clinical samples. Nat Protoc.

[13] Mbala-Kingebeni P., Villabona-Arenas C.J., Vidal N., Likofata J., Nsio-Mbeta J., Makiala-Mandanda S. (2019). Rapid confirmation of the Zaire Ebola virus in the outbreak of the equateur province in the democratic republic of congo: implications for public health interventions. Clin Infect Dis.

[14] Schmieder R., Edwards R. (2011). Quality control and preprocessing of metagenomic datasets. Bioinformatics.

[15] Nurk S., Bankevich A., Antipov D., Gurevich A.A., Korobeynikov A., Lapidus A. (2013). Assembling single-cell genomes and mini-metagenomes from chimeric MDA products. J Comput Biol.

[16] Safonova Y., Bankevich A., Pevzner P.A. (2015). dipSPAdes: assembler for highly polymorphic diploid genomes. J Comput Biol.

[17] Buchfink B., Xie C., Huson D.H. (2015). Fast and sensitive protein alignment using DIAMOND. Nat Methods.

[18] Ondov B.D., Bergman N.H., Phillippy A.M. (2011). Interactive metagenomic visualization in a web browser. BMC Bioinform.

[19] Doan T., Pinsky B.A. (2016). Current and future molecular diagnostics for ocular infectious diseases. Curr Opin Ophthalmol.

[20] Brown J.R., Morfopoulou S., Hubb J., Emmett W.A., Ip W., Shah D. (2015). Astrovirus VA1/HMO-C: an increasingly recognized neurotropic pathogen in immunocompromised patients. Clin Infect Dis.

[21] Fischer N., Rohde H., Indenbirken D., Gunther T., Reumann K., Lutgehetmann M. (2014). Rapid metagenomic diagnostics for suspected outbreak of severe pneumonia. Emerg Infect Dis.

[22] Greninger A.L., Chen E.C., Sittler T., Scheinerman A., Roubinian N., Yu G. (2010). A metagenomic analysis of pandemic influenza A (2009 H1N1) infection in patients from north America. PLoS One.

[23] Greninger A.L., Naccache S.N., Federman S., Yu G., Mbala P., Bres V. (2015). Rapid metagenomic identification of viral pathogens in clinical samples by real-time nanopore sequencing analysis. Genome Med.

[24] Loman N.J., Constantinidou C., Christner M., Rohde H., Chan J.Z., Quick J (2013). A culture-independent sequence-based metagenomics approach to the investigation of an outbreak of shiga-toxigenic Escherichia coli O104:H4. JAMA.

[25] Lum S.H., Turner A., Guiver M., Bonney D., Martland T., Davies E. (2016). An emerging opportunistic infection: fatal astrovirus (VA1/HMO-C) encephalitis in a pediatric stem cell transplant recipient. Transpl Infect Dis.

[26] Naccache S.N., Peggs K.S., Mattes F.M., Phadke R., Garson J.A., Grant P. (2015). Diagnosis of neuroinvasive astrovirus infection in an immunocompromised adult with encephalitis by unbiased next-generation sequencing. Clin Infect Dis.

[27] Pallen M.J. (2014). Diagnostic metagenomics: potential applications to bacterial, viral and parasitic infections. Parasitology.

[28] McCarthy C.B., Diambra L.A., Rivera Pomar R.V. (2011). Metagenomic analysis of taxa associated with lutzomyia longipalpis, vector of visceral leishmaniasis, using an unbiased high-throughput approach. PLoS Negl Trop Dis.

[29] Jacobs M., Rodger A., Bell D.J., Bhagani S., Cropley I., Filipe A. (2016). Late ebola virus relapse causing meningoencephalitis: a case report. Lancet.

[30] Naccache S.N., Peggs K.S., Mattes F.M., Phadke R., Garson J.A., Grant P. (2015). Diagnosis of neuroinvasive astrovirus infection in an immunocompromised adult with encephalitis by unbiased next-generation sequencing. Clin Infect Dis.

[31] Frey K.G., Herrera-Galeano J.E., Redden C.L., Luu T.V., Servetas S.L., Mateczun A.J. (2014). Comparison of three next-generation sequencing platforms for metagenomic sequencing and identification of pathogens in blood. BMC Genom.

[32] Quick J., Grubaugh N.D., Pullan S.T., Claro I.M., Smith A.D., Gangavarapu K. (2017). Multiplex PCR method for MinION and Illumina sequencing of Zika and other virus genomes directly from clinical samples. Nat Protoc.

[33] Schlagenhauf P., Weld L., Goorhuis A., Gautret P., Weber R. (2015). Travel-associated infection presenting in Europe (2008–12): an analysis of EuroTravNet longitudinal, surveillance data, and evaluation of the effect of the pre-travel consultation. Lancet Infect Dis.

[34] Khairat R., Ball M., Chang C.C., Bianucci R., Nerlich A.G., Trautmann M (2013). First insights into the metagenome of Egyptian mummies using next-generation sequencing. J Appl Genet.

[35] Klein R.S. (2008). A moving target: the multiple roles of CCR5 in infectious diseases. J Infect Dis.

[36] Wei X., Nielsen R. (2019). CCR5-32 is deleterious in the homozygous state in humans. Nat Med.

