# Supplementary material for: Metagenomic next-generation sequencing aids the diagnosis of viral infections in febrile returning travellers
Source: J Infect. 2019 Oct;79(4):383–8. doi: 10.1016/j.jinf.2019.08.003 (PMC6859916; doi:10.1016/j.jinf.2019.08.003)
Supplement: Supplementary file 1 [file mmc1.docx]

**Supplementary Figure 1: MNGS** **diagnostic thresholds**

As MNGS is subject to cross-contamination, a threshold of <10% genome coverage was validated with an alternative test. When two samples were identified as positive on the same sequencing run, or the coverage was <10%, the sample with the lower coverage required a confirmatory test e.g. repeat MNGS or diagnostic PCR or serology.


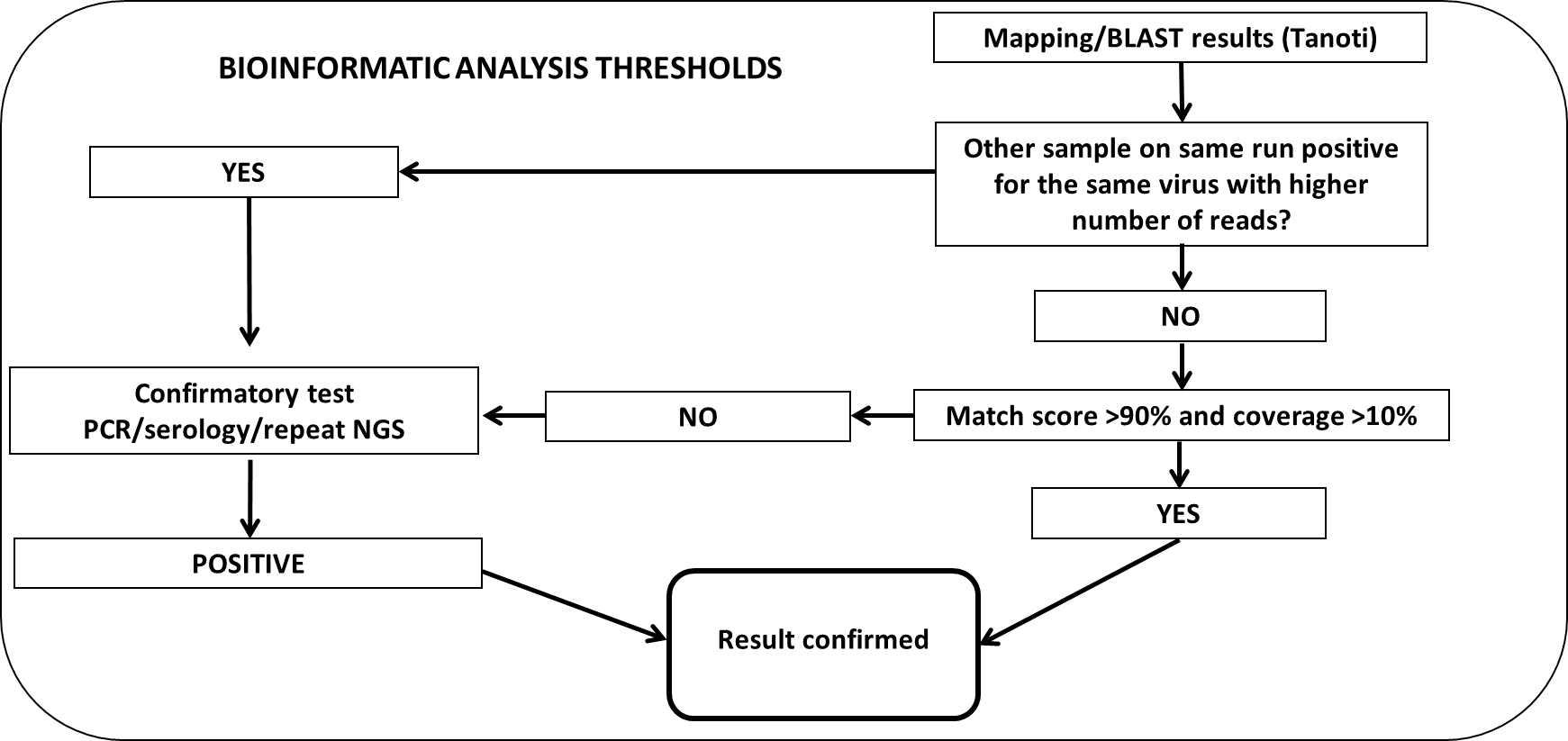


**Supplementary figure 2: Phylogenetic analysis of HPgV sequences**

A maximum likelihood phylogenetic tree was created using the General Time Reversible (GTR) model and 1000 bootstrap replicates, using 3 near-full genome sequences and 7 reference sequences representing the 6 putative genotypes of HPgV and one unassigned genotype.

**
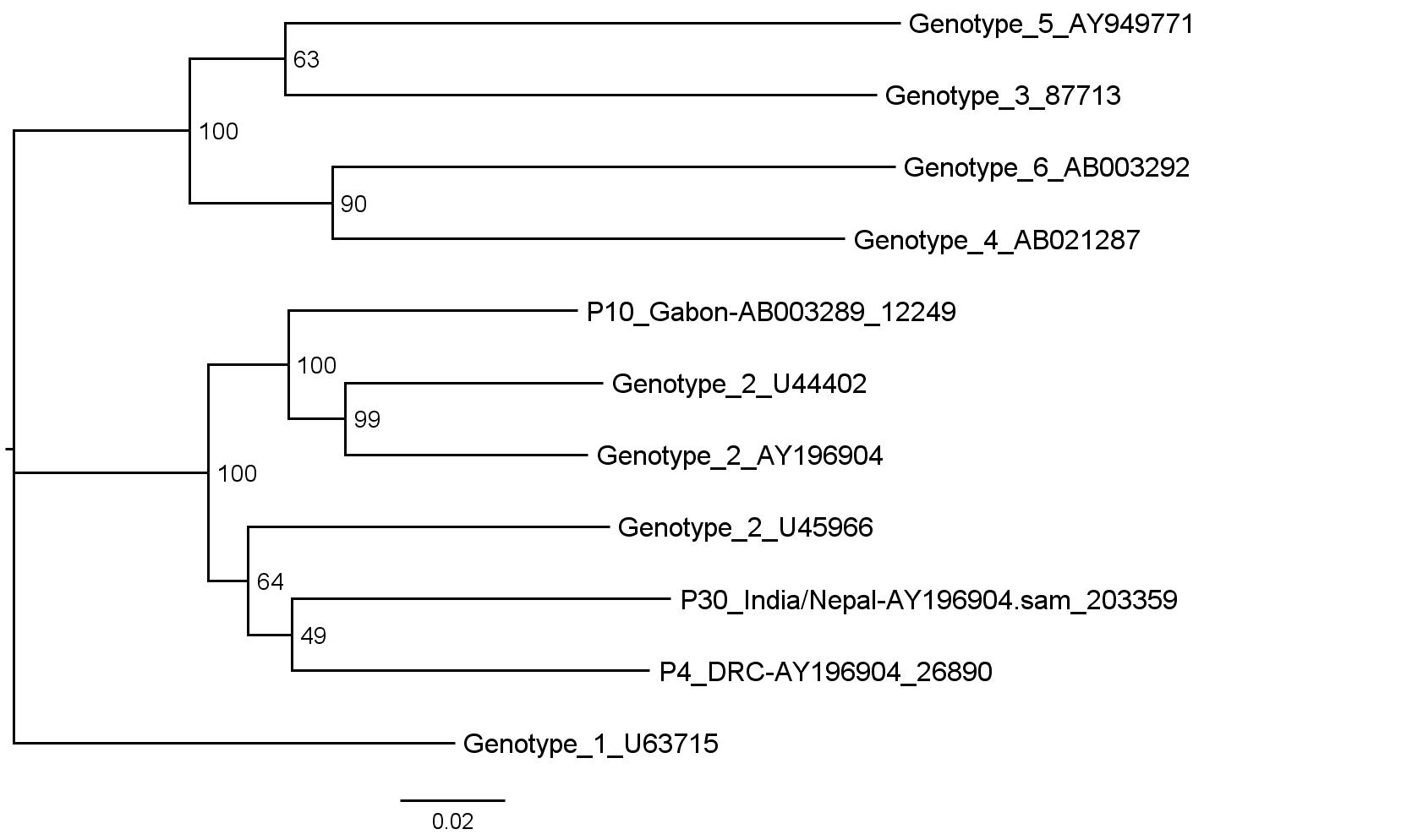
**

| Sample | Nearest reference (NS3 region) | p-distance (amino acid) |
| --- | --- | --- |
| P10 Gabon | Genotype 2 AY196904 | 0.06 |
| P30 India/Nepal | Genotype 2 U45966 | 0.00 |
| P4 DRC | Genotype 2 U45966 | 0.18 |
|  |  |  |
| Sample | **Nearest reference (NS5B region)** | **p-distance (amino acid)** |
| P10 Gabon | Genotype 2 AY196904 | 0.29 |
| P30 India/Nepal | Genotype 2 AY196904 | 0.14 |
| P4 DRC | Genotype 2 AY196904 | 0.36 |

**Supplementary Table 1:** **Travel history and clinical laboratory diagnosis**

| Patient | Sample | Travel history | Pathogen (clinical laboratory) | Viral/parasite NGS |
| --- | --- | --- | --- | --- |
| 1 | LRT1 | Vietnam | Positive Rickettsial serology (RIPL) | None |
| 2 | LRT2 | Equatorial Guinea | *Plasmodium malariae* (blood film) | *Plasmodium malariae (18S)* |
| 3 | LRT3 | Kenya | *Plasmodium falciparum* (blood film) | *Plasmodium falciparum (18S)* |
| 4 | LRT4 | DRC | None detected | HPgV (highly divergent genotype 2) |
| 5 | LRT5 | Pakistan | None detected | None |
| 6 | LRT6 | USA | None detected | None |
| 7 | LRT7 | India | None detected | None |
| 8 | LRT8 | Namibia, South Africa | None detected | None |
| 9 | LRT9 | Mexico | *Salmonella oranienburg* (blood culture) | None |
| 10 | LRT10 | Gabon | *Streptococcus pneumoniae* (culture of aortic tissue) | HPgV (genotype 2) |
| 11 | LRT11 | India | Hepatitis E virus PCR | HEV |
| 12 | LRT12 | India | Influenza A (throat swab PCR) | None in serum  Throat swab not tested |
| 13 | LRT13 | Uganda | Influenza B (throat swab PCR) | None in serum  Throat swab not tested |
| 14 | LRT14 | Mexico | Control sample (afebrile traveller) | None |
| 15 | LRT15 | Mexico | None detected | None |
| 16 | LRT16 | Cameroon | None detected | None |
| 17 | LRT17 | Maldives | Dengue virus (PCR and serology) | DENV1 (Sri Lanka) |
| 18 | LRT18 | Indonesia | None detected (dengue suspected clinically) | Chikungunya virus (Asian strain) |
| 19 | LRT19 | Unknown | None detected | None |
| 20 | LRT20 | India | Salmonella (culture of stool sample); cause of fever/hepatitis undetected | None |
| 21 | RT1 | Papua New Guinea | *Plasmodium falciparum* (blood film), severe | *Plasmodium falciparum (18S)* |
| 22 | RT2 | Somalia | Acute Hepatitis A Infection (IgM positive on serology) | HAV |
|  | RT19 |  |  | HAV |
| 23 | RT3 | Vietnam, Laos, Cambodia | None detected | None |
| 24 | RT4 | Thailand | *Plasmodium falciparum* (blood film), mild | None |
| 25 | RT5 | Ghana | None detected | None |
| 26 | RT6 | Sri Lanka | None detected | None |
| 27 | RT7 | Peru | None detected | None |
| 28 | RT8 | Malaysia | None detected | None |
| 29 | RT9 | South Sudan | *Plasmodium falciparum* (low parasitemia) | *Plasmodium falciparum (18S)* |
| 30 | RT11 | India, Nepal | Rhinovirus (PCR) | HPgV (Genotype 2) |
| 31 | RT10 | United Arab Republic & Pakistan | Invasive helminth infection | None |
| 32 | RT12 | Unknown | None detected | None |
| 33 | RT13 | Majorca | None detected (cholangitis) | None |
| 34 | RT14 | Ghana | *Plasmodium falciparum* (blood film) |  |
|  | RT15 |  |  | Mumps virus |
|  | RT16 |  |  |  |
| 35 | RT17 | Thailand | Dengue virus (PCR) | DENV1 (Thailand/China) |
|  | RT22 |  |  | DENV1 (Thailand/China) |
| 36 | RT18 | Kenya, Rwanda, Uganda, DRC | None detected | None |
| 37 | RT20 | Florida | None detected | None |
| 38 | RT21 | India, Pakistan | None detected | None |
| 39 | RT23 | Uganda | *Plasmodium malariae* (antigen test) | None |
| 40 | RT24 | Cameroon | None detected | Chikungunya |
| 41 | RT25 | Sierra Leone | EBOV (Zaire strain, PCR) | EBOV (Zaire) |
